# Association of acute hepatitis B and acute myopathy: a case report

**DOI:** 10.1186/s13256-022-03330-w

**Published:** 2022-04-09

**Authors:** Mehrangiz Zangeneh, Masoomeh Mesgarian, Yasamin Khosravani-Nezhad

**Affiliations:** 1 Department of Infectious Diseases, Faculty of Medicine, Tehran Medical Sciences, Islamic Azad University, Zargandeh st, Khaghani st, Shariati, Tehran, Iran; 2 Reproductive Biomedicine Research Center, Royan Institute for Reproductive Biomedicine, ACECR, Tehran, Iran; 3 Islamic Azad University of Medical Sciences, Zargandeh st, Khaghani st, Shariati, Tehran, Iran

**Keywords:** Hepatitis B, Myopathy, Acute

## Abstract

**Background:**

Hepatitis B virus infection is a global public health problem. Although hepatitis B virus primarily affects hepatocytes, it sometimes develops disease manifestations outside the liver, such as myopathy, which is commonly caused by chronic hepatitis B.

**Case presentation:**

This case report describes a 57-year-old Iranian woman admitted to the hospital with jaundice, fever, body itching, abdominal pain, progressive muscle weakness, icteric sclera, right upper quadrant pain, and decreased muscle force. Examination on the first day of admission revealed that the patient was negative for hepatitis D antibody, positive for hepatitis B core (IgM) antibody, positive for hepatitis B surface antigen, and negative for hepatitis B e antibody but positive for hepatitis B e antigen. Moreover, she showed high levels of hepatitis B virus DNA viral load, creatine kinase, lactate dehydrogenase, serum glutamic-oxaloacetic transaminase, serum glutamic-pyruvic transaminase, total bilirubin, direct bilirubin, and alkaline phosphatase, and electromyography/nerve conduction velocity showed acute myopathic process.

**Conclusions:**

Interestingly, myopathy symptoms improved after improving hepatitis symptoms and decreasing hepatitis B viral load, suggesting a close association between hepatitis B infection and myopathy.

## Background

Hepatitis B virus (HBV) infection is a global public health problem. It is estimated that there are 248 million HBV carriers globally, of whom roughly 600,000 die annually from HBV-related liver disease. The overall prevalence of HBsAg is reported to be 3.6%; however, prevalence varies depending upon the geographic area. The majority of chronic HBV ranges from < 2% in low-prevalence regions (for example, USA, Canada, Western Europe) to 2–7% in intermediate-prevalence areas (for example, Mediterranean countries, Japan, Central Asia, Middle East, and parts of South America) and ≥ 8% in high-prevalence areas (for example, Western Africa, South Sudan). HBV can be transmitted from mother to child, via transfusion, sexually, percutaneously (injection drug users, contaminated instruments, piercings), or via transplant [1, 2].

The spectrum of clinical manifestations of HBV infection varies in both acute and chronic diseases. During the acute phase, manifestations range from subclinical or anicteric hepatitis to icteric hepatitis and, in some cases, fulminant hepatitis. Manifestations vary from an asymptomatic carrier state to chronic hepatitis, cirrhosis, and hepatocellular carcinoma during the chronic phase.

A serum sickness-like syndrome in acute icteric hepatitis B may develop during the prodromal period, followed by constitutional symptoms, anorexia, nausea, jaundice, and right upper quadrant discomfort. The signs and jaundice generally disappear after 1–3 months, but some patients have prolonged fatigue even after normalization of serum aminotransferase concentrations [3]. Hepatitis B infection may have extrahepatic manifestations, but most are related to chronic hepatitis B infection. Rarely, acute HBV infection can be associated with extrahepatic conditions. Extrahepatic manifestations include serum sickness-like syndrome, polyarthritis, polyarteritis nodosa, dermatologic conditions (bullous pemphigoid, lichen planus, Gianotti–Crosti syndrome), cryoglobulinemia (common: Raynaud phenomenon, arthritis, sicca syndrome; less common: renal, neurologic, or vasculitic complications), and neurologic/psychological conditions (Guillain–Barré syndrome, altered mental status, depression/psychosis) [4]. The pathophysiology of these associated symptoms is mainly based on complex immune reactions in the skin, joints, muscles, and kidneys [5]. We could not find in the literature the relation between acute hepatitis B and acute myopathy. Systemic diseases that cause myopathy include metabolic disorders as well as inflammatory, paraneoplastic, endocrine, drug- and toxin-induced diseases or infection. Among infectious etiologies, viral infections most commonly cause myopathy, such as influenza, parainfluenza, Coxsackie, human immunodeficiency virus (HIV), cytomegalovirus, echovirus, adenovirus, and Epstein–Barr virus, and several animal parasites, such as protozoa (*Toxoplasma*, *Trypanosoma*), cestodes (cysticerci), and nematodes (trichinae), may produce a focal or diffuse inflammatory myopathy known as parasitic polymyositis. Though less common, *Staphylococcus aureus*, *Yersinia*, *Streptococcus*, or other anaerobic bacteria like clostridia may cause supportive myositis or Lyme myositis, and, in immunocompromised patients, fungal myositis may be caused by, for example, sporotrichosis, histoplasmosis, mucormycosis, cryptococcosis, or candidiasis [6]. Neuropathy develops in approximately 5% of patients with chronic HBV infection and rarely during acute HBV infection. Rare cases of muscle disease, mainly inflammatory myopathy, have been reported with chronic HBV infection [7, 8]. In the present case report, we, for the first time, describe a case with acute hepatitis B coexisting with acute myopathy.

## Case presentation

A 57-year-old Iranian female was referred to the infectious diseases department at Amiralmomenin Medical School hospital with jaundice, fever, body itching, abdominal pain, progressive muscle weakness, and pain from 1 week ago; on physical examination, she was found to have icteric sclera, right upper quadrant pain, and reduced muscle force. The patient had a history of diabetes mellitus (10 years), hypothyroidism, hyperlipidemia, hypertension, and diabetic foot 5 months ago. The patient’s drug history included atorvastatin [40 mg per oral (PO) every day (QD), acetylsalicylic acid (80 mg PO QD], losartan (25mg PO QD), ranitidine (150 mg PO every 12 hours), amlodipine (2.5 mg PO QD), and furosemide (40 mg PO QD). The patient has been using insulin for the past 2 years. The patient was admitted to the hospital with the clinical diagnosis of acute hepatitis and myopathy. Before acute hepatitis, the patient did not have serum creatine kinase (CK) elevation due to statin consumption. On neurological examination, at the time of admission, muscle strength was 4 out of 5 in upper limbs and 2 out of 5 in lower limbs. One day after being hospitalized, the patient developed urinary retention leading to catheterization. Then, the muscle weakness became so severe that the patient was unable to walk and do defecation. After 1 week, the patient was visited by a neurologist and rheumatologist. All joints were normal, but limb muscle force and strength of gluteal muscle and external anal sphincter decreased. Initial differential diagnoses were acute hepatitis, statin toxicity, and rhabdomyolysis. As reported in the tables, the patient’s lab results demonstrated that the virology test for HBV Ag was positive with a high viral load level; however, the tests for other hepatitis viruses and HBV Ab were negative (Table 1). At the first visit, lab tests demonstrated an increase in aminotransferases (ALT, AST), alkaline phosphatase, bilirubin, CK, lactate dehydrogenase (LDH), aldolase, and ferritin; however, the results improved 14 days later without any specific medication (Table 2).

**Table 1:** Results of lab tests

WBC	11,000/mm^3^	Hbs Ag (ELISA)	Positive
RBC	327 × 10^4^/mm^3^	HBe Ag	Positive
Platelet count	327,000	HBc Ab	Positive
HGB	10.5 g/dl	HBc Ab IgM	Positive > 15 IU/ml
ESR	77 → 32 mm	HBe Ab	Negative
C-reactive protein	10 mg/dl	HDV Ab	Negative
Urea	52 mg/dl	HCV Ab	Negative
Creatinine	1.2 mg/dl	HIV Ab	Negative
Fe	85 mg/dl	HBs Ab	Negative
TIBC	282	HEV	Negative
Calcium	8.5 mg/dl	HAV Ab	Negative
Phosphorus	3.3 mg/dl	HBV viral load	3,319,701,150 Copies/ml 570,395,386 IU/mL
Prothrombin time (PT)	12.5 seconds		
Ferritin	960 ng/ml → 357 ng/ml		
Potassium	3.6 mEq/l	Rheumatoid factor	Negative
Blood culture	Negative	ANA	Negative
Wright	Negative	p-ANCA	Negative
Coombs Wright	Negative	c-ANCA	Negative
Troponin	Negative	Anti-ds-DNA	Negative
Venereal disease research laboratory (VDRL)	Negative	Antimitochondrial Ab	Negative
Thyroxine (T4)	13 μg%	Anti-smooth muscle Ab	Negative
Triiodothyronine (T3)	1.5 ng/ml	Tissue transglutaminase Ab	Negative
Thyroid-stimulating hormone (TSH)	3.4 mlU/ml	Anti-gliadin Ig G	Negative
Copper in a normal range	In blood and urine	Anti-gliadin IgA	Negative
Total protein	6.3 mg/dl	LKM-1	Negative
Albumin	3.4 mg/dl	Anti-endomysial Ab	Negative
Urinalysis	Normal	Aldolase	40 IU/l
Chest x-ray	Normal	Ceruloplasmin	31 mg/dl, normal

HGB, Hemoglobin; ESR, Erythrocyte Sedimentation Rate; Fe, Iron; TSH, Thyroid-Stimulating Hormone; Hbs, Hepatitis B Surface; Hbc, Hepatitis B Core; HDV, Hepatitis D Virus; HCV, Hepatitis C Virus; HEV, Hepatitis E Virus; HAV, Hepatitis A Virus; HBV, Hepatitis B Virus; Ag, Antigen; Ab, Antibody; RF, Rheumatoid Factor; ANA, Antinuclear Antibodies; C-ANCA, Antineutrophil Cytoplasmic Autoantibody Cytoplasmic; P-ANCA, Perinuclear Anti-Neutrophil Cytoplasmic Antibodies; Ig, Immunoglubolin; →, shows the recovery process

**Table 2:** Electromyography (EMG)/nerve conduction velocity (NCS) results

Neuron site	Nerve	Muscle	Result
Sensory NCS	L sural—Lat malleolus	L calf	Not obtainable
	R sural—Lat malleolus	R calf	Not obtainable
	L superficial peroneal—foot	L Lat leg	Not obtainable
Motor NCS	Tibial malleolus—abductor hallucis brevis	Wrist	Not obtainable
		Elbow	Not obtainable
	L common peroneal—extensor digitorum brevis (EDB)	Ankle	Not obtainable
		Fibular head	Not obtainable
	R common peroneal—EDB	Ankle	Not obtainable
		Fibular hand	Not obtainable
H-Reflex	L tibial—soleus	Knee	Not obtainable
	R tibial—soleus	Knee	Not obtainable
EMG	L TIB Ant		Mildly decreased
	L gastrocnemius—Lat		Mildly decreased
	L vastus lateralis		Early recruitment
	R vastus lateralis		Early recruitment
	R biceps		Early recruitment
	R deltoid		Early recruitment

Summary: (1) assessment motor and sensory nerve conduction was not possible at every neuron site; (2) on needle EMG examination, low-amplitude motor unit potentials (MUPs) with early recruitment were most prominent in proximal muscles of upper and lower limbs. Conclusion: The patient was found to exhibit (1) acute myopathic process and (2) chronic sensorimotor polyneuropathy. L, left; R, right; Lat, lateral; TIB, tibial; Ant, anterior

An ultrasonogram of the abdomen showed mild hepatomegaly and splenomegaly. Urine analysis (U/A) was negative for myoglobin. Electromyography/nerve conduction velocity (EMG/NCV) revealed acute myopathic process and chronic sensorimotor polyneuropathy (Table 2). Chronic sensorimotor polyneuropathy was a complication of diabetes mellitus. All rheumatologic tests were negative, including antinuclear antibody (ANA), peripheral antineutrophil cytoplasmic antibody (p-ANCA), cytoplasmic antineutrophil cytoplasmic autoantibody (c-ANCA), anti-double-stranded (ds)-DNA, antimitochondrial Ab, anti-smooth muscle Ab, tissue transglutaminase Ab, antigliadin Ab (IgG, IgM), and liver kidney microsomal type 1 (LKM-1). Copper (urine and blood), Fe, total iron binding capacity (TIBC), and ceruloplasmin were in normal range; Wright, Coombs Wright, and blood culture were negative. Urine analysis for myoglobinuria was negative, ruling out rhabdomyolysis.

Without any treatment, the patient’s hepatitis and muscle strength improved gradually. She was able to walk with a walker on discharge day after improving hepatitis symptoms, liver function test, and decreasing HBV viral load. The patient was discharged on day 19 with a relatively good general condition. The final diagnosis at discharge was coexisting acute hepatitis B and myopathy as a complication of viral hepatitis B (Table 3).

**Table 3:** Laboratory results

	At first visit	3 days later	7 days later	10 days later	14 days later
CK, IU/l	11,200	4605	3359	830	207
LDH, IU/l	1250	882	842	636	823
SGOT, IU/l	1161	763	529	225	371
SGPT, IU/l	970	785	603	335	334
Billi T, mg/dl	3.7	4.4	2.6	3.7	3.4
Billi D, mg/dl	3.5	2.9	2.3	1.3	1.3
ALK P, IU/l	1172	1033	842	878	877
Na, mg/dl	121	118	116	131	137

CK, Creatine Kinase; LDH, Lactate Dehydrogenase; SGPT, Serum Glutamic Pyruvic Transaminase; SGOT, Serum Glutamic-Oxaloacetic Transaminase; T, Total; D, Direct

Outpatient follow-up on day 30 and 1 year later revealed complete recovery, with the following test results: serum glutamic-oxaloacetic transaminase (SGOT) 13 IU/l, serum glutamic-pyruvic transaminase (SGPT) 25 IU/l, alkaline phosphatase (ALK P) 230 IU/l, creatine kinase (CK) 152 IU/l, LDH 589 IU/l, total bilirubin (Billi T) 0.9 mg/dl, direct bilirubin (Billi D) 0.2 mg/dl, HBS Ag negative, HBS Ab 171 mIU/mL.

## Discussion

There are some case reports on the association between HBV and muscle disease, but most are chronic HBV and myositis, not myopathy. Bernard *et al*. reported on a patient who developed myalgia associated with mild hepatitis B surface antigen-positive hepatitis. Muscle biopsy showed myriads of microvacuoles filled with neutral lipid (lipid storage myopathy). Prednisone therapy was associated with complete clearing of all clinical and laboratory abnormalities [7]. Flatau ^et al^. reported on a 38-year-old man complaining of progressive weakness, muscle pains, impotence, and weight loss with chronic hepatitis B virus (HBV)-positive liver disease, like in our case but with acute hepatitis [9]. Capasso *et al*. showed the association of chronic hepatitis B and myopathy in two cases. In these cases, they revealed that the pathogenesis of myopathy is an immune-mediated mechanism. Infected muscle fibers bearing viral antigens could be the target of an immune response leading to necrosis. In their patients, some isolated or grouped fibers were positive for viral antigens, suggesting that infection itself could have induced major histocompatibility complex (MHC)-I expression and that viral antigens co-expressed with MHC-I, making infected fibers the target of an immune response. The improvement of patients during antiviral therapy also suggests a direct role of HBV in myopathy, but we did not perform a muscle biopsy in our case. Still, with the improvement of hepatitis, the muscle weakness disappeared [10]. Stübgen describes rare cases of muscle disease, mainly inflammatory myopathy, associated with HBV infection in a review article. He explained that, presumably, HBV-associated antigens trigger immune mechanisms directed against components of muscle tissue [8]. In a case report by Dr. Thanapirom in Thailand, a 56-year-old male was admitted to the hospital with chronic HBV cirrhosis, hepatocellular carcinoma (HCC), and polymyositis. As Dr. Thanapirom explained, a possible cause for this association was an autoimmune process by CD8-positive cytotoxic T cells that could induce the release of MHC class 1 antigen and cytokines in muscle fibers, causing inflammation and leading to polymyositis [11]. The pathogenesis of the acute HBV-associated myopathy in this case report is not clear, though it might involve deposition of immune complexes in muscle fiber or blood vessel walls, or direct viral infection of muscle fiber. We did not perform muscle biopsy to assess whether the pathogenesis of myopathy was direct viral infection or immune mediated. Because the myopathy improved when HBV viral load was reduced gradually, we think the pathogenesis is direct invasion of virus antigen to components of muscle fibers.

## Conclusion

Hepatitis B infection can trigger the immune system and cause immune-mediated syndromes such as peripheral neuropathy and myopathy or directly invade muscle fibers and cause myopathy.

## Data Availability

All the information used in this manuscript is available in the patient file at the center.
